# Room temperature tunable multicolor phosphorescent polymers for humidity detection and information encryption†

**DOI:** 10.1039/d2ra00294a

**Published:** 2022-03-14

**Authors:** Yulei Gao, Xiang Di, Fenfen Wang, Pingchuan Sun

**Affiliations:** Key Laboratory of Functional Polymer Materials of Ministry of Education and College of Chemistry, State Key Laboratory of Medicinal Chemical Biology, Nankai University Tianjin 300071 P. R. China wff@mail.nankai.edu.cn spclbh@nankai.edu.cn

## Abstract

Amorphous polymer-based room temperature phosphorescence (RTP) materials exhibiting tunable emission colors have received tremendous attention and are extremely challenging to prepare. Herein, polyacrylamide-based RTP materials with tunable multicolor emission were prepared *via* copolymerizing phosphor with concentration dependent luminescence colors and acrylamide with different molar ratios. The hydrogen bonding interactions and chemically crosslinked structures in these polymers effectively restrict the mobility of phosphors and activate efficient RTP emission. The molar ratio of phosphor and acrylamide has a significant influence on the photophysical properties of these polymers, which can be used to fabricate multicolor materials. In addition, the RTP intensity decreases with increasing humidity due to the disassociation of hydrogen bonding by adsorption of water, manifesting as a humidity sensor. Benefiting from the distinguishable RTP lifetimes and the responsiveness to humidity, triple encoding for information encryption is successfully realized.

## Abstract 2

A polymer with tunable multicolor was prepared *via* copolymerizing a phosphor with concentration dependent luminescence and acrylamide based on chemical crosslinking and hydrogen bonding interactions for humidity detection and information encryption.

## Graphical abstract

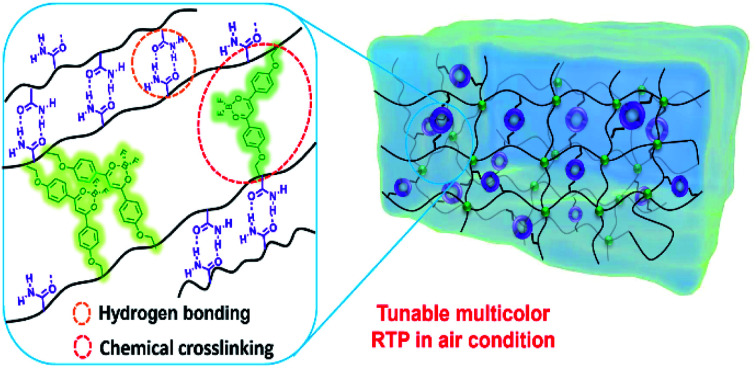

## 1. Introduction

Room temperature phosphorescence (RTP) materials have become a research spotlight of chemists and materials scientists with their potential applications in the field of sensors,^1,2^ organic light-emitting diodes (OLED),^3^ bioimaging^4^ and document security^5^ owing to their unique photophysical properties. Generally, the strategies for designing efficient RTP materials include promoting the intersystem crossing (ISC) from the lowest excited singlet state (S_1_) to the excited triplet state (T_*n*_), suppressing the nonradiative transition from the lowest excited triplet state (T_1_) to the ground state (S_0_) and shielding oxygen effectively which can easily quench T_1_. Conventional RTP materials are mainly coordination complexes containing noble metals such as platinum and palladium which can enhance spin–orbit coupling and facilitate the ISC process. However, the development of novel metal-free RTP materials is necessary due to drawbacks such as expensive, relatively toxic and limited resources for organometallic compounds. Based on the microscopic mechanism of RTP generation, several approaches have been put forward to prepare pure organic RTP materials, including crystallization-induced,^6–10^ embedding phosphors in a rigid matrix^11,12^ and H-aggregation^13,14^*etc.* The nonradiative deactivation of triplet excitons is maximally suppressed due to the rigidity and oxygen barrier properties of crystals, while the complex growth process and poor processability limit their commercial applications. The past decade has witnessed the extensive investigation of polymer-based RTP materials owing to low cost, easy processing and low toxicity compared with crystalline RTP materials and coordination complexes containing noble metals.^15–22^ For example, Kim and co-workers devised Diels–Alder click chemistry as a method to covalent cross-linking between phosphors and polymer matrices to suppress molecular motions for enhanced RTP of metal-free organic materials.^23^ Tian and co-workers developed a facile way to construct pure organic amorphous polymers with high phosphorescent quantum yields and ultralong RTP lifetimes by the simple radical binary copolymerization of acrylamide and different phosphors without extra processing.^24,25^ Lu *et al.* proposed a reliable large-scale strategy for synthesizing the covalently efficient RTP materials *via* B–O click reaction between boronic acid-modified phosphors and polyhydroxy polymer matrix.^26^ Tang *et al.* presented wide-color ranged and persistent RTP from amorphous films by embedding electron-rich organic phosphor into electron-deficient matrix polyacrylonitrile.^27^ Despite numerous advances, developing advanced RTP materials still remains challenging in several aspects, for example, most of polymer-based RTP materials just emit a single color. If the luminescent color can vary with external stimulation, it will effectively expand the application range of materials and meet the new development requirements of organic functional materials.^28,29^

Color-tunable luminescent materials, especially phosphorescence emission, have attracted tremendous interests due to their potential applications in sensing, information encryption and anti-counterfeiting.^30,31^ For instance, Zhao *et al.* achieved color-tunable ultralong RTP in single polymer through radical multicomponent luminophores cross-linked copolymerization.^32^ Besides, amorphous pure organic copolymers with multicolor RTP emission have been prepared by Zhang *et al. via* copolymerizing two phosphors (benzoic acid and 4-bromo-1,8-naphthalic anhydride) and acrylamide with different feeding ratios.^33^ Furthermore, multicolor phosphorescence signals were also observed in the doped binary luminescent copolymer systems.^34^ However, the incorporation of phosphors with divergent emission colors in a single polymer to generate multicolor RTP emission will complicate the luminescence system and make it difficult to control the homogeneity of each phosphor in polymer matrix.^35^ Accordingly, designing and developing single-phosphor RTP materials with multicolor luminescence for further broadening and optimizing the tunable RTP emission systems is highly desired.

Luminescence color tuning is essentially the alteration of the transition energy levels of the phosphor molecules.^36^ Research reveals that for certain polar phosphors, different phosphor–phosphor interactions regulated by concentration can affect the ground state or excited state energies of light-emitting cores, which analogous to solvatochromism.^37^ This implies that covalent bonding of phosphors with concentration-dependent luminescent colors into a polymer matrix may be a reasonable strategy to prepare color-tunable RTP emission materials. Difluoroboron β-diketonates (BF_2_bdks) are a classic polar fluorescent dyes and have emerged as a class of functionalized luminescent materials because of their large extinction coefficient, sensitivity to the surrounding environment, high quantum yields and chemical stability.^38,39^ After Fraser's group first reported the RTP of BF_2_bdks induced by coupling with poly(lactic acid),^40^ emission color tuning for RTP materials based on BF_2_bdks has been investigated, while persistent RTP is restricted to inert conditions which limits the application of BF_2_bdks.^41–44^

**Scheme 1:**
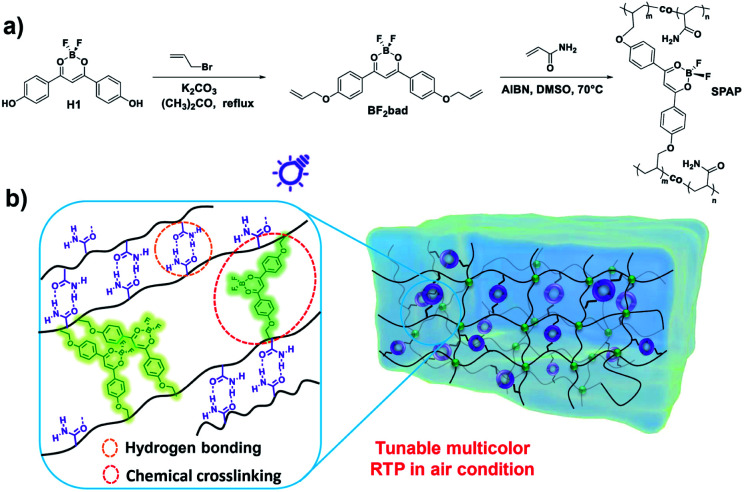
(a) Synthetic routes of BF_2_bad and SPAP. (b) Rational design strategy of tunable multicolor RTP in SPAP.

Herein, we proposed a new strategy to prepare a single-phosphor amorphous polymer (abbreviated as SPAP) with efficient RTP emission under ambient conditions *via* combining BF_2_bdks (named as BF_2_bad) and acrylamide monomer (Scheme 1a). Where the chemical crosslinking structures and high-density hydrogen bonds between polyacrylamide (PAM) chains are devised as the effective method to restrict molecular motion (Scheme 1b) of BF_2_bdks and polymer chains. It is demonstrated that the molar ratio of two comonomers (BF_2_bad and acrylamide) has a significant effect on the photophysical properties of SPAP which can be used for fabricating tunable multicolor materials. Besides, since hydrogen bonding interactions can be broken by water, the moisture content is quantitatively detected by the change in RTP emission intensity, and information encryption is also realized.

## 2. Experimental

### 2.1 Materials

Methyl 4-methoxybenzoate, 4′-methoxyacetophenone, potassium carbonate (K_2_CO_3_) and sodium hydride (NaH, 60% dispersed in mineral oil) were supplied by Aladdin, while magnesium sulfate (MgSO_4_), boron tribromide (BBr_3_) and 3-bromopropene were obtained from Innochem. Boron trifluoride-diethyl etherate (BF_3_·Et_2_O) was purchased from Shanghai Merrill Chemical Technology Co., Ltd. Acrylamide and 2,2′-azobis(2-methylpropionitrile) (AIBN) were obtained from TCI. AIBN was recrystallized from anhydrous ethanol before use. All solvents were commercially available and used as received.

### 2.2 Synthesis of difluoroboron-1,3-bis(4-(allyloxy)phenyl)propane-1,3-dione (BF_2_bad)

Under magnetic stirring, difluoroboron-1,3-bis(4-hydroxyphenyl)propane-1,3-dione (H1, 1.0 g, 3.29 mmol, see Scheme S1 (ESI†) for the synthetic route), 3-bromopropene (1.49 g, 12.35 mmol) and K_2_CO_3_ (1.136 g, 8.22 mmol) were added into acetone (20 mL), and the reaction mixture was heated to 60 °C for 10 h (monitored by TLC). After cooled to room temperature, the deionized water (20 mL) was added and extracted with CH_2_Cl_2_ (3 × 20 mL). The organic phase was collected, dried with MgSO_4_, filtered and concentrated under reduced pressure to yield the BF_2_bad as a yellow solid (1.07 g, 84.9% yield). ^1^H NMR (Fig. S4 (ESI†), 400 MHz, in CDCl_3_): *δ* 8.11 (d, 4H, H_a_), 7.03 (d, 4H, H_b_), 7.01 (s, 1H, H_c_), 6.06 (ddd, 2H, H_d_), 5.45 (d, 2H, H_e_), 5.36 (d, 2H, H_e’_), 4.66 (d, 4H, H_f_); ^13^C NMR (Fig. S5 (ESI†), 101 MHz, in CDCl_3_, *δ* ppm): 180.93, 164.45, 132.20, 131.36, 124.75, 118.73, 115.36, 91.69, 69.31.

### 2.3 Typical procedures for the preparation of SPAP

Taking polymer SPAP(1/50) as a typical example, SPAP(1/50) was prepared by free radical copolymerization of compound BF_2_bad (1 eq.) and acrylamide (50 eq.) by AIBN (1.4 wt% of acrylamide) as radical initiator at 70 °C under nitrogen atmosphere in 4.5 mL DMSO for 17 h. After cooled to room temperature, the mixture was added dropwise to methanol to precipitate and the resulting solid was washed 3–5 times with methanol, to give a SPAP(1/50).

### 2.4 Instruments and methods

#### 2.4.1 NMR experiments

^1^H NMR and ^13^C NMR spectra were recorded on a Bruker AVANCE III spectrometer in CDCl_3_ or acetone-d_6_ with chemical shifts reported as ppm.

#### 2.4.2 Powder X-ray diffraction (XRD)

XRD was performed on a Japan SmartLab 9 KW spectrometer at a scanning rate of 3° s^−1^.

#### 2.4.3 Scanning electron microscopy (SEM)

The morphologies of SPAP were observed by SEM (QUANTA 200, FEI).

#### 2.4.4 Ultraviolet-visible spectroscopy (UV-vis)

UV-vis spectra were done on a Shimadzu UV-2450 spectrophotometer.

#### 2.4.5 Photoluminescence measurements

Photoluminescence measurements (for solution) were performed on a Hitachi Fluoremax-4600 spectrophotometer. Photoluminescence spectra, lifetimes (for solid) were obtained on a FS5 (Edinburgh Analytical Instruments Ltd.) spectrophotometer with a xenon lamp or a microsecond flashlamp as excitation light source (*λ*_ex_ = 365 nm; delay time = 0.1 ms). Quantum yields were measured by using an integrating sphere on a HAMAMATSU C9920-02. Photographs were taken with a digital camera.

#### 2.4.6 Estimation of H_2_O detection limit

A plot of the phosphorescence quenching efficiency measured at 365 nm *versus* the volume fraction of water allowed to calculate the limit of detection (LOD) according to the formula LOD = 3*σ*/*k*, where *σ* is the standard deviation for the blank solution, which was measured eleven times, and *k* denotes the slope of the curve.^45^

## 3. Results and discussion

### 3.1 Preparation of SPAP with different molar ratios

The copolymerization between phosphors and polymer matrix is identified as a common method to achieve RTP under ambient conditions by suppressing thermal motions and shielding quenchers.^20,25,46^ The abundant hydrogen bonds in the PAM can further effectively immobilize phosphors and greatly enhance the RTP emission intensity, which prompts us to explore whether the fluorescent dye BF_2_bdks with multifaceted optical properties can also inflict RTP in this system. Therefore, SPAP was synthesized through thermal initiated radical copolymerization of BF_2_bad and acrylamide. The characterizations of ^1^H NMR and ^13^C NMR are described in the supplementary information† (Fig. S4 and S5, ESI†). To evaluate the influence of the molar ratio of BF_2_bad and acrylamide on the photophysical properties of materials (*e.g.*, emission wavelength and phosphorescence lifetime), SPAPs were synthesized whose molar ratios of BF_2_bad and acrylamide were 1/10, 1/25, 1/50, 1/75 and 1/100, respectively (Table S1, ESI†).

### 3.2 Photophysical properties of BF_2_bad

**Fig. 1:**
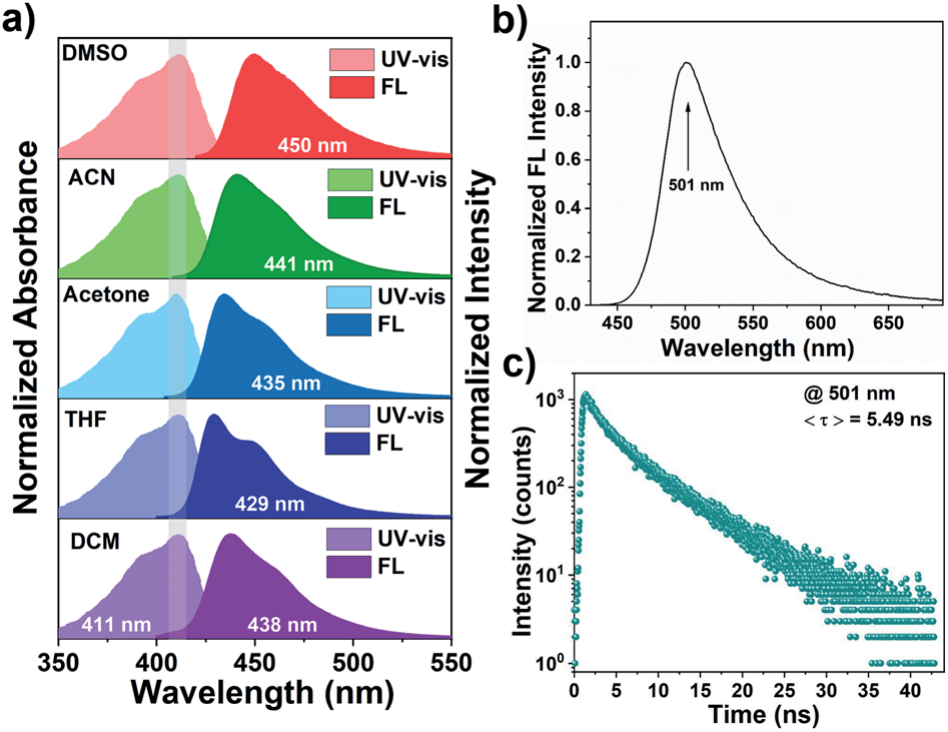
(a) Normalized UV-vis absorption and fluorescence emission spectra of BF_2_bad in different solvents (10 μM, *λ*_ex_ = 411 nm). (b) Normalized steady-state fluorescence emission spectrum of BF_2_bad in the solid state (*λ*_ex_ = 365 nm). (c) Time-resolved photoluminescence decay of BF_2_bad in the solid state.

Photophysical properties of monomer BF_2_bad in different solvents with varied polarities were first investigated by UV-vis absorption and fluorescence spectra. As shown in Fig. 1a, the maximum absorption peak of BF_2_bad in the diluted dichloromethane solution was observed at 411 nm with a molar extinction coefficient as high as 69 700 M^−1^ cm^−1^ (Fig. S6, ESI†), which was ascribed to the typical π–π* transitions.^47^ The higher extinction coefficient indicates that BF_2_bad has a stronger light-harvesting ability compared with other BF_2_bdks.^48,49^ Besides, due to the electron-donating ability of allyloxy substituent and the electron-accepting ability of difluoroboron β-diketone moieties, BF_2_bad also exhibited an intramolecular charge-transfer (ICT) transition, which could be supported by the solvent polarity-dependent fluorescence emission spectra.^50,51^ As we can see in Fig. 1a, the absorption spectra were slightly influenced as increasing solvent polarity (dichloromethane < tetrahydrofuran < acetone < acetonitrile < dimethyl sulfoxide), while the emission of BF_2_bad manifested positive solvatochromism. For example, the emission spectra showed a red shift from 429 nm in tetrahydrofuran to 450 nm in dimethyl sulfoxide. In addition, owing to the interaction between molecules, the maximum emission wavelength bathochromic shifted to 501 nm in the solid state with a fluorescence lifetime of 5.49 ns (Fig. 1b and c). The above-mentioned results demonstrate that BF_2_bad is an excellent stimuli-responsive phosphor and can be used for the preparation of color-tunable luminescent materials, which will be further discussed subsequently. However, due to the quenching effect and nonradiative relaxation process, phosphorescence emission was not observed for BF_2_bad monomer in either solution or solid state.

### 3.3 Multicolor fluorescence and RTP of SPAP

**Fig. 2:**
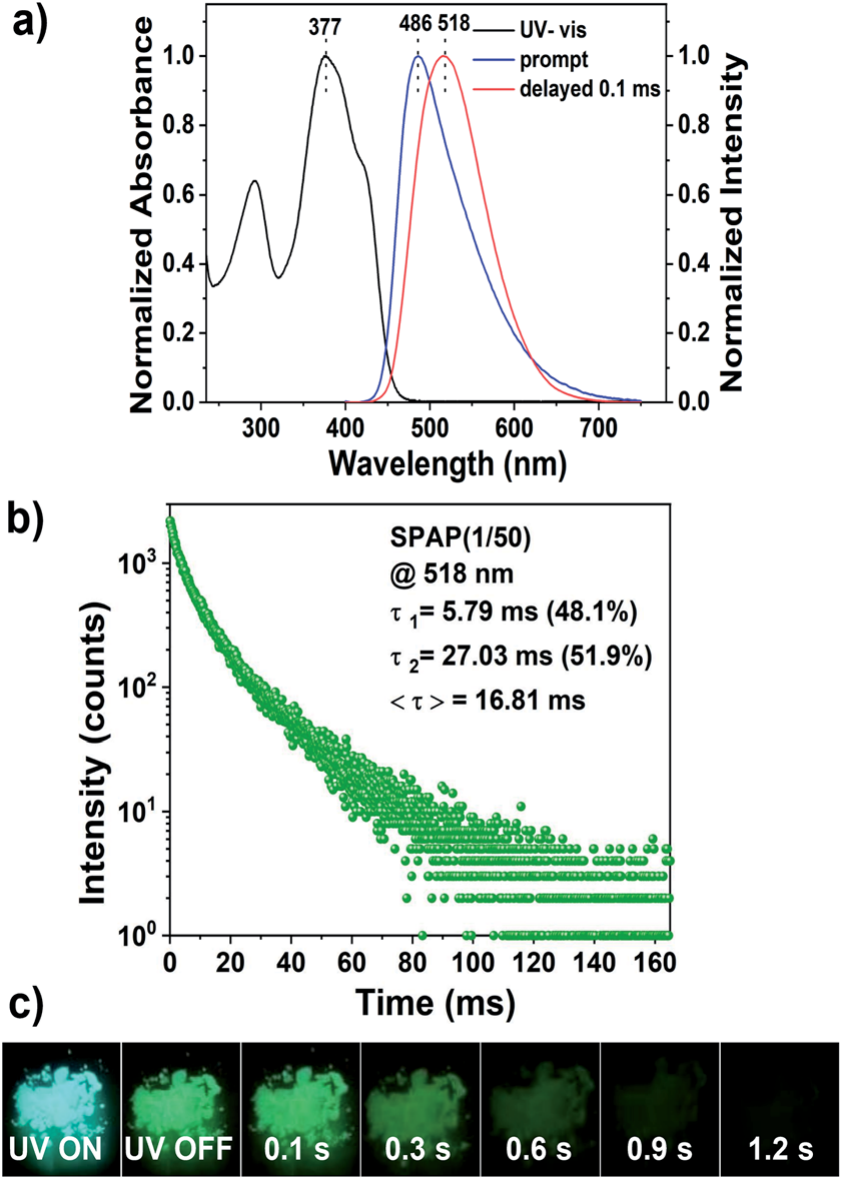
(a) Normalized UV-vis absorption, prompt and delayed photoluminescence spectra of SPAP(1/50). (b) Lifetime decay curves of SPAP(1/50) at 518 nm. (c) Luminescence photographs of SPAP(1/50) under 365 nm UV irradiation and after ceasing the UV irradiation.

After copolymerization with acrylamide, SPAP(1/50) (BF_2_bad : acrylamide = 1 : 50, molar ratio) was obtained. SPAP(1/50) exhibited green persistent phosphorescence at 518 nm with a lifetime of 16.8 ms after turning off the UV irradiation under ambient conditions, as shown in Fig. 2a and b. The afterglow could be observed by the naked eye (Fig. 2c). Moreover, larger Stokes shift (approximately 140 nm) and red-shifted emission peak compared to fluorescence emission (*λ*_em_ = 486 nm) further indicate that the delayed luminescence is derived from phosphorescence rather than possible delayed fluorescence.

**Fig. 3:**
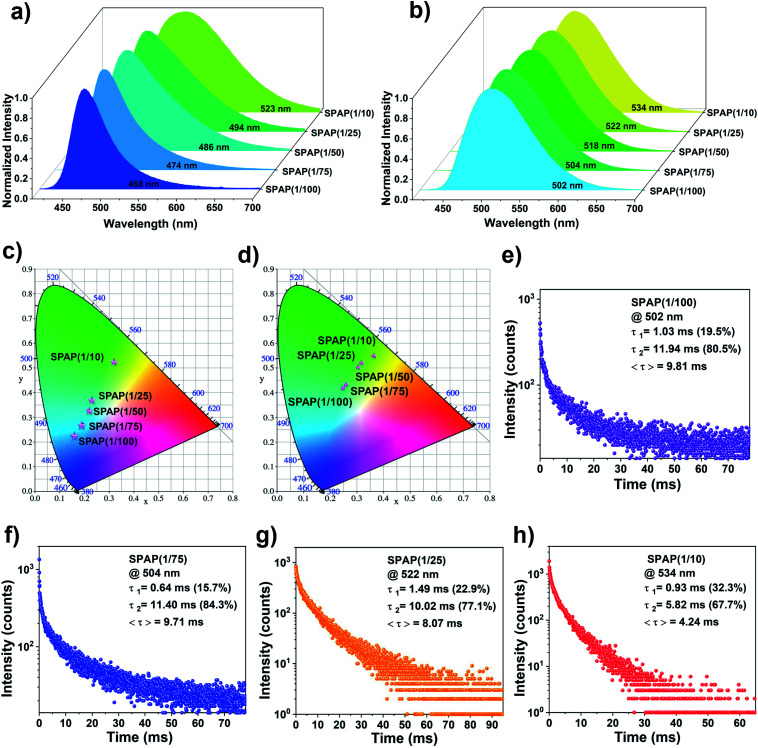
Normalized (a) steady-state fluorescence and (b) RTP spectra of SPAP (*λ*_ex_ = 365 nm, delay time = 0.1 ms). CIE 1993 coordinates of the (c) fluorescence emission and (d) RTP emission of SPAP. (e–h) Lifetime decay curves of SPAP whose molar ratios are 1/100, 1/75, 1/25 and 1/10, respectively.

The fluorescence emission and RTP emission spectra of SPAP were further investigated (Fig. 3a and b) and the corresponding photophysical data was summarized in Table 1. In general, solvatochromism is related to the difference in the polarity of the surrounding liquid or solid-state medium;^52^ however, the polarity of the medium may also be influenced by polar dyes.^53^ In terms of materials containing BF_2_bdks, this is reflected in the different phosphor loadings.^44,54^ Clearly, just as the fluorescence emission of BF_2_bad red-shifted with increasing solvent polarity, the fluorescence emission wavelength of SPAP also showed a bathochromic shift from 468 nm (SPAP(1/100)) to 523 nm (SPAP(1/10)) as the BF_2_bad content increased which accompanied by the luminescence color turned from blue to green under environment conditions, and the spectra became broader. The RTP emission wavelength of SPAP showed the similar trend as the fluorescence emission. The corresponding Commission Internationale de l'Éclair-age (CIE) coordinates were calculated in Fig. 3c and d. According to previous reports, BF_2_bdks have a larger dipole moment^38^ and their emission is sensitive to the polarity of the surrounding medium.^55^ Higher concentration of BF_2_bad will result in a greater probability of BF_2_bad–BF_2_bad interactions in the system. When the distance between BF_2_bad molecules is about 3 Å,^56,57^ ground–state association or excimers will be formed, which resulting in lower energy emission.^37^ Conversely, the reduction of BF_2_bad–BF_2_bad interactions is responsible for the short-wave emission at lower BF_2_bad content. The results indicate that varying the feed amount of BF_2_bad is a simple but efficient approach to generate color tunability for PAM-based RTP systems.

**Table 1:** Photophysical properties of SPAP

Sample	*λ*_F_ [nm]	*λ*_P_ [nm]	*τ*_P_ [ms]	*Φ*_F_ [%]	*Φ*_P_ [%]
SPAP(1/100)	468	502	9.81	30.27	3.90
SPAP(1/75)	474	504	9.71	32.87	3.29
SPAP(1/50)	486	518	16.81	33.33	2.31
SPAP(1/25)	494	522	8.07	31.39	4.11
SPAP(1/10)	523	534	4.24	22.04	3.24

Furthermore, both phosphorescence lifetime (*τ*_P_) and phosphorescence quantum yield (*Φ*_P_) of SPAP were also measured and calculated (Table 1 and Fig. 3e–h). *τ*_P_ and *Φ*_P_ are important physical quantities to describe the properties of phosphorescence, among which, *τ*_P_ refers to the time required for the phosphorescent intensity to decay to 1/*e* of the initial value. *Φ*_P_ is a measure of the utilization efficiency of absorbed photons during the phosphorescent process, and is defined as the ratio of the number of emit ted phosphorescent photons and the number of absorbed photons. The equations are shown below:

1 *τ*_P_ = 1/(*k*_P_ + *k*_ts_ + *k*_q_)

2 *Φ*_P_ = *Φ*_isc_*k*_P_/(*k*_P_ + *k*_ts_ + *k*_q_)

where *Φ*_isc_ is the rate constant of intersystem crossing, *k*_P_ represents the rate constant of phosphorescence emission, *k*_ts_ refers to the nonradiative transition rate constant from T_1_ to S_0_ and *k*_q_ is the rate constant of quenching *T*_1_ state by quenchers.

From eqn (1), the key to obtaining long *τ*_P_ is to minimize *k*_ts_ and *k*_q_. The abundant hydrogen bonding interactions among the PAM chains restrict the molecular motion of phosphors associated with *k*_ts_ and effectively shield quenchers like oxygen associated with *k*_q_.^25^ Accordingly, longer *τ*_P_ can be observed at lower content of BF_2_bad. In contrast, higher BF_2_bad content will reduce the number of hydrogen bonds and weaken the shielding effect of quenchers, thus is thought to cause a shorter *τ*_P_. As shown in Table 1, *τ*_P_ shortens from 16.8 ms (SPAP(1/50)) to 4.2 ms (SPAP(1/10)) as the BF_2_bad content increases. Even so, very low contents will also lead to a shortening of *τ*_P_ because enough BF_2_bad contact is necessary to achieve RTP.^17,58,59^ Besides, *Φ*_P_ varies slightly with the molar ratio of SPAP, which is influenced by the competition between phosphorescence emission and other nonradiative relaxations, such as vibrational dissipation and oxygen-mediated quenching.^17,18,60^

To verify the important role of copolymerization of BF_2_bad and acrylamide for realizing RTP emission, we tested the delayed photoluminescence spectra of mechanical mixture of BF_2_bad and PAM (SPAP′(1/50)) as a control experiment. As shown in Fig. S8 (ESI†), SPAP′(1/50) has no emission peak at 518 nm, indicating that simple mixing cannot bind BF_2_bad to PAM tightly and thus the thermal motion of BF_2_bad cannot be inhibited effectively. As mentioned above, the copolymerization of BF_2_bad with acrylamide is the key to active RTP emission. The abundant hydrogen bonding interactions and the inherent crosslinked structure in SPAP can effectively promote RTP emission by locking phosphors and shielding quenchers.

### 3.4 Microstructure characterization of SPAP

**Fig. 4:**
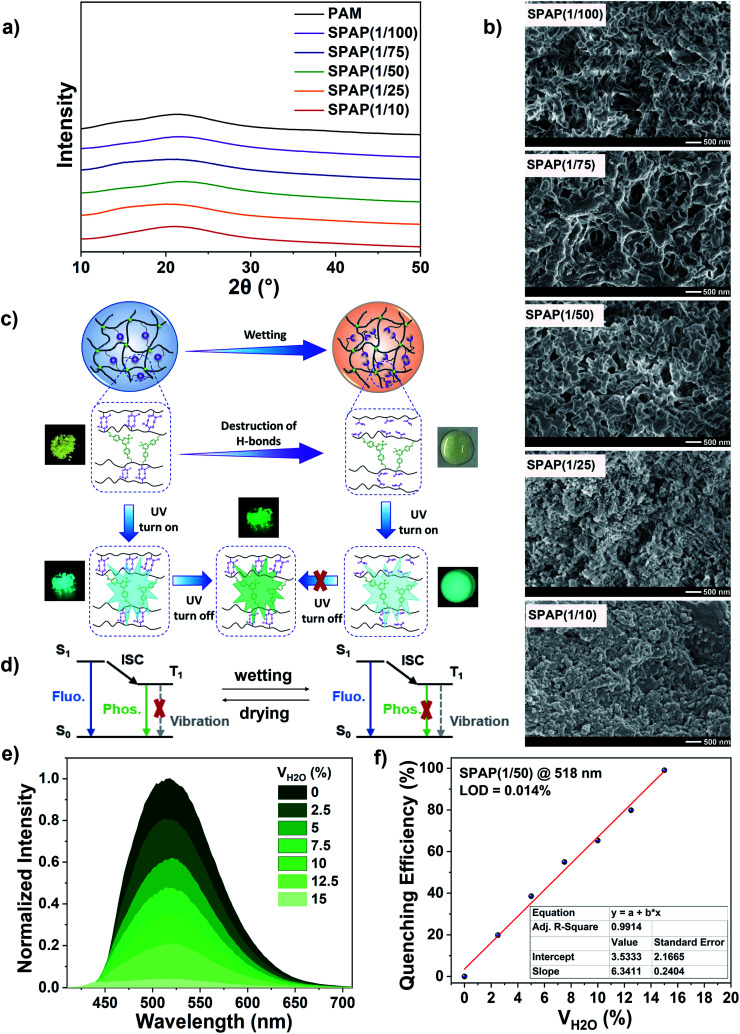
(a) XRD spectra of PAM and SPAP. (b) SEM images of SPAP. (c) Schematic illustration and (d) Jablonski diagram of RTP quenching due to hydrogen bonds destruction. (e) Normalized RTP emission spectra of SPAP(1/50) in H_2_O/DMF mixed solutions (*c* = 1.5 mg mL^−1^). (f) The dependence of phosphorescence quenching efficiency of SPAP(1/50) at 518 nm on water fraction, the red line is the linear fitting curve.

Since the microstructure of polymers, such as crystalline or amorphous state, have a great influence on the corresponding luminescence properties,^24,25,46^ XRD analysis was carried out to determine the structure of SPAP, among which PAM was also studied as a reference polymer. As shown in Fig. 4a, PAM was amorphous from the only broad characteristic diffraction peak appeared at 2*θ* = 20–23°. SPAP also showed an amorphous structure similar to PAM, indicating that the amorphous state of these polymers is related to their luminescence properties.

The microscopic morphology of SPAP also was observed by SEM and the corresponding porosity was estimated (Fig. S9, ESI†). As shown in Fig. 4b, SPAP(1/100) showed a distinct porous network structure which became progressively denser with increasing BF_2_bad content (see SPAP(1/10)). Understandably, the chemical crosslinking density of SPAP increased as the phosphor content increased due to the modification of the end group double bond functionalization which made BF_2_bad act as a chemical crosslinker, resulting in a more dense microscopic morphology. Meanwhile, the denser structure of SPAP promoted BF_2_bad–BF_2_bad interactions and facilitated the formation of ground-state association or excimers, which was consistent with previous results.

### 3.5 Humidity responsiveness of RTP intensity

The rigid environment consisting of chemical crosslinking and interchain interactions *via* hydrogen bonding can immobilize BF_2_bad, shield quenchers and enhance the RTP emission. Therefore, we speculated that the presence of water would reduce the rigidity of polymer network thus weaken the RTP emission intensity of SPAP to a certain extent because the hydrogen bonding interactions can be broken by water (Fig. 4c and d). Since PAM is only soluble in water, we prepared seven suspension samples of 1.5 mg mL^−1^ by dispersing SPAP(1/50) in H_2_O/DMF mixed solutions to explore the effect of humidity on the RTP emission intensity. As shown in Fig. 4e, the RTP emission intensity of SPAP(1/50) at 518 nm gradually decreased with increasing the volume fraction of water, and was totally quenched when the water fraction reached 15%. It is noteworthy that the phosphorescence quenching efficiency followed a good linear correlation with water content (*R*^2^ = 0.9914) (Fig. 4f) and the LOD was calculated as 0.014%, which is comparable to other fluorescent sensors for water detection,^61^ which makes SPAP(1/50) a potential candidate for moisture content detection in organic solvents.

### 3.6 Information encryption application

**Fig. 5:**
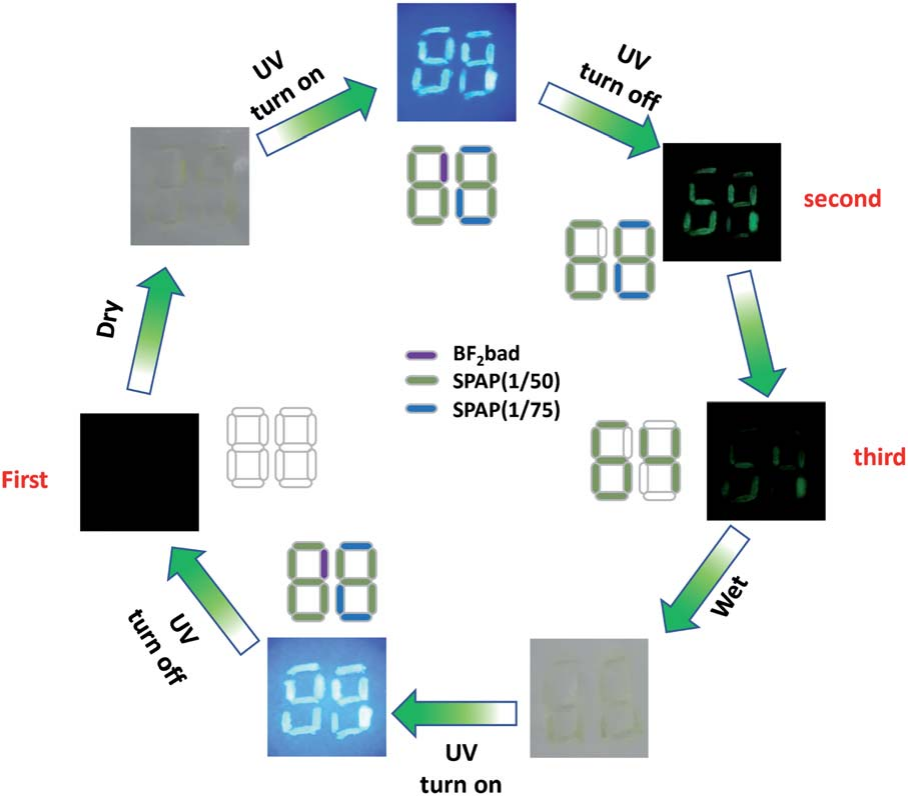
Photographs of triple encoding for digit encryption.

Benefiting from the diversified RTP lifetimes and the humidity responsiveness, these polymers can be applied to multiple encodings for information encryption. As an example shown in Fig. 5, the digits were written by different inks in a piece of non-fluorescent paper, where the number “64” was written by long-lived SPAP(1/50) (dissolved in water, 20 mg mL^−1^), the blue part of “88” was marked by SPAP(1/75) (dissolved in water, 20 mg mL^−1^) and the purple part was coated with BF_2_bad (dissolved in dichloromethane, 20 mg mL^−1^). Number “88” could be found under the daylight in both the wet and dry state due to the fluorescence properties of BF_2_bad. In the dry state, the bright number “88” appeared under the irradiation of UV lamp and disappeared immediately after turning off the irradiation due to no RTP emission of BF_2_bad, leaving the long-lived SPAP(1/50) and SPAP(1/75) emitted a green luminescence of “68”. Thenceforth, the RTP of SPAP(1/75) decayed to invisibility and the residual phosphorescence of SPAP(1/50) emitted “64” (Movie 2, ESI†). Under moist state, number “88” was still visible under UV light, but nothing could be found after turning off the UV light due to the quenching of RTP. As a result, the triple encoding of digital encryption was realized, and this process was completely reversible and could be repeated more than once.

## 4. Conclusions

In summary, we have successfully designed and synthesized concentration-dependent luminescent metal-free amorphous RTP materials with color-tunable through radical copolymerization of the single phosphor BF_2_bad and acrylamide. The results indicate that the chemical crosslinking structures and the hydrogen bonding interactions between polymer chains effectively restrict the molecular motion of phosphors and shield the quenching of the triplet excitons by oxygen, creating the necessary conditions for observing the persistent RTP with the naked eye under environmental conditions. Multicolor phosphorescence signals are observed in this polymer system which can be effectively regulated by changing the feed amount of phosphor. The phosphorescence quenching mechanism is attributed to the disassociation of hydrogen bonds in the presence of water. Furthermore, triple encoding of information encryption is achieved by utilizing the humidity response and the different RTP lifetimes of SPAP. This work is expected to have applications in the fields of moisture content detection and document security as well as will further contribute to the development of tunable multicolor materials.

## Author contributions

Yulei Gao: conceptualization, data curation, writing original draft, and visualization. Xiang Di: methodology and investigation. Fenfen Wang: conceptualization, resources, writing – review & editing. Pingchuan Sun: resources, writing – review & editing, supervision, project administration, and funding acquisition.

## Conflicts of interest

The authors declare no conflict of interest.

## Supplementary Material

RA-012-D2RA00294A-s001

RA-012-D2RA00294A-s002

RA-012-D2RA00294A-s003
