# Supplementary material for: Room temperature tunable multicolor phosphorescent polymers for humidity detection and information encryption
Source: RSC Adv. 2022 Mar 14;12(13):8145–53. doi: 10.1039/d2ra00294a (PMC8982396; doi:10.1039/d2ra00294a)
Supplement: RA-012-D2RA00294A-s001 [file RA-012-D2RA00294A-s001.pdf]

Electronic Supplementary Material (ESI) for RSC Advances.
This journal is © The Royal Society of Chemistry 2022

Supplementary Information

Room temperature tunable multicolor phosphorescent polymers for humidity detection and information encryption

Yulei Gao,[a] Xiang Di,[a] Fenfen Wang[a,*] and Pingchuan Sun[a,*]

[a]Key Laboratory of Functional Polymer Materials of Ministry of Education and College of Chemistry, State Key Laboratory of Medicinal Chemical Biology, Nankai University, Tianjin 300071, China
E-mail: wff@mail.nankai.edu.cn; spclbh@nankai.edu.cn

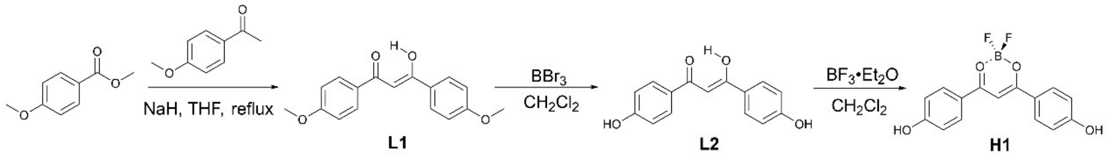

Scheme S1. Synthetic route of H1.

Compound 1,3-bis(4-methoxyphenyl)propane-1,3-dione (L1) was synthesized according to the method which has been reported in the earlier literature with same structure.[1]

Synthesis of 1,3-bis(4-hydroxyphenyl)propane-1,3-dione (L2): To a solution of L1 (3.46 g, 12.2 mmol) in CH_2Cl_2 (50 mL) cooled with an ice-salt bath was added dropwise a 1 M CH_2Cl_2 solution of BBr_3 (36.5 mL, 36.5 mmol). This solution was stirred for 30 min at ice-salt bath, 30 min at 0 °C, 5 h at room-temperature and afterward quenched by dropwise addition of H_2O (50 mL). Addition of 2 M NaOH (50 mL). The aqueous was adjusted to pH = 6 with conc HCl (aq) caused formation of a yellow precipitate which was then extracted with ethyl acetate 3 times, and the combined organic phase was washed with brine (20 mL), dried ($MgSO_4$), filtrated and concentrated under vacuum to afford L2 as a yellow solid (2.77 g, 88.8% yield). ^{1}H NMR (400 MHz, in

Acetone-d_6): δ 17.58 (s, 0.9H), 9.12 (s, 2H), 8.04 (d, 4H), 7.04 (s, 1H), 6.97 (d, 4H), 4.60 (s, 0.1H).

Synthesis of difluoroboron-1,3-bis(4-hydroxyphenyl)propane-1,3-dione (H1): To a solution of L2 (2.56 g, 10.0 mmol) in CH_2Cl_2 (50 mL) added dropwise $BF_3 \cdot Et_2O$ (2.5 mL, 20.0 mmol) by a syringe. The mixture was heated slowly to 60 °C for 5.5 h under nitrogen atmosphere. After cooled to room temperature, the mixture was concentrated under reduced pressure. The desire pure product was obtained by recrystallization from ethyl acetate as a yellow solid (2.18 g, 71.7% yield). ^1H NMR (400 MHz, in Acetone-d_6): δ 9.69 (s, 1.5H), 8.25 (d, 4H), 7.51 (s, 1H), 7.06 (d, 4H).

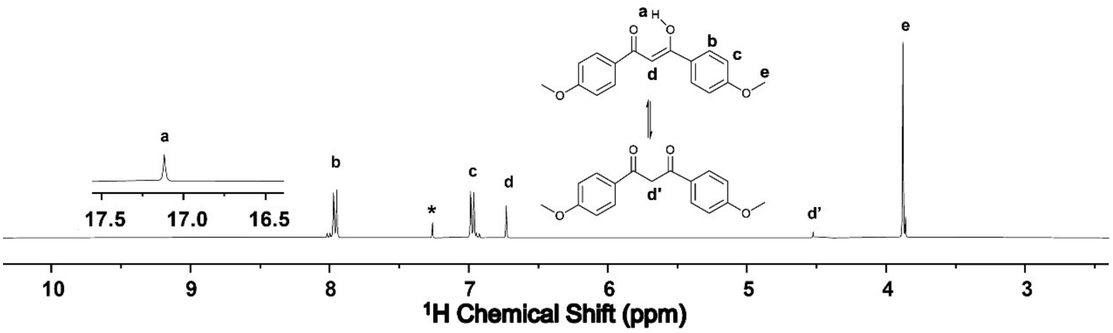

Figure S1. The ^1H NMR spectrum of L1 in $CDCl_3$.

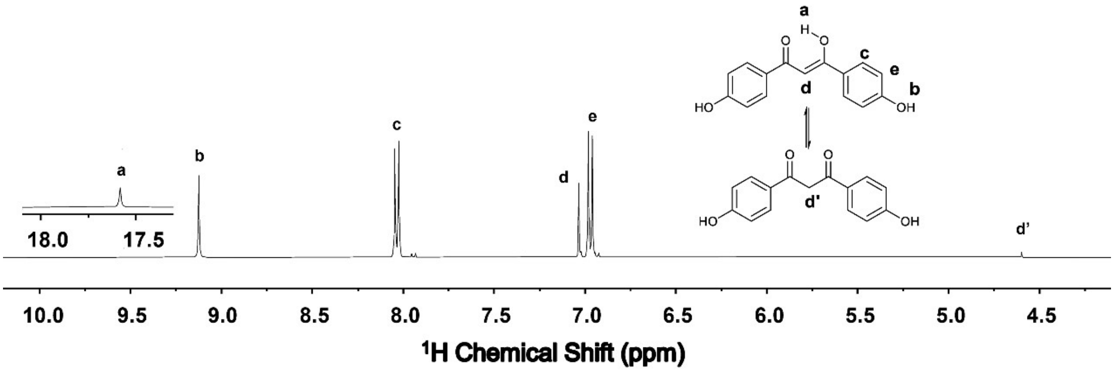

Figure S2. The ^1H NMR spectrum of L2 in Acetone-d_6.

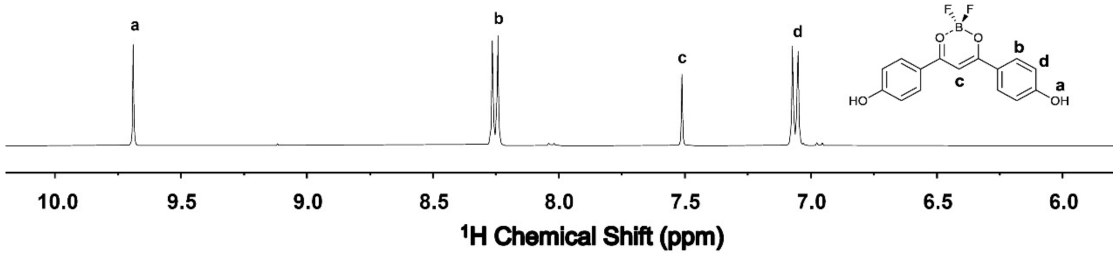

Figure S3. The ^{1}H NMR spectrum of H1 in Acetone-d_6.

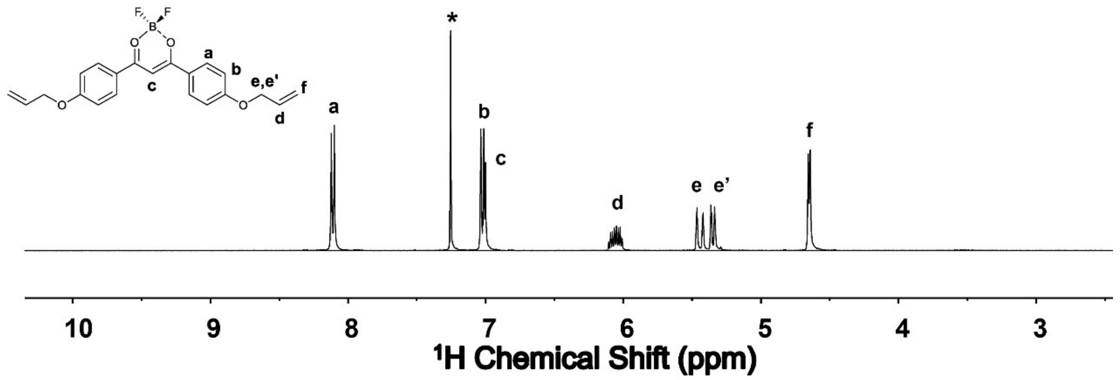

Figure S4. The ^{1}H NMR spectrum of BF_2bad in $CDCl_3$.

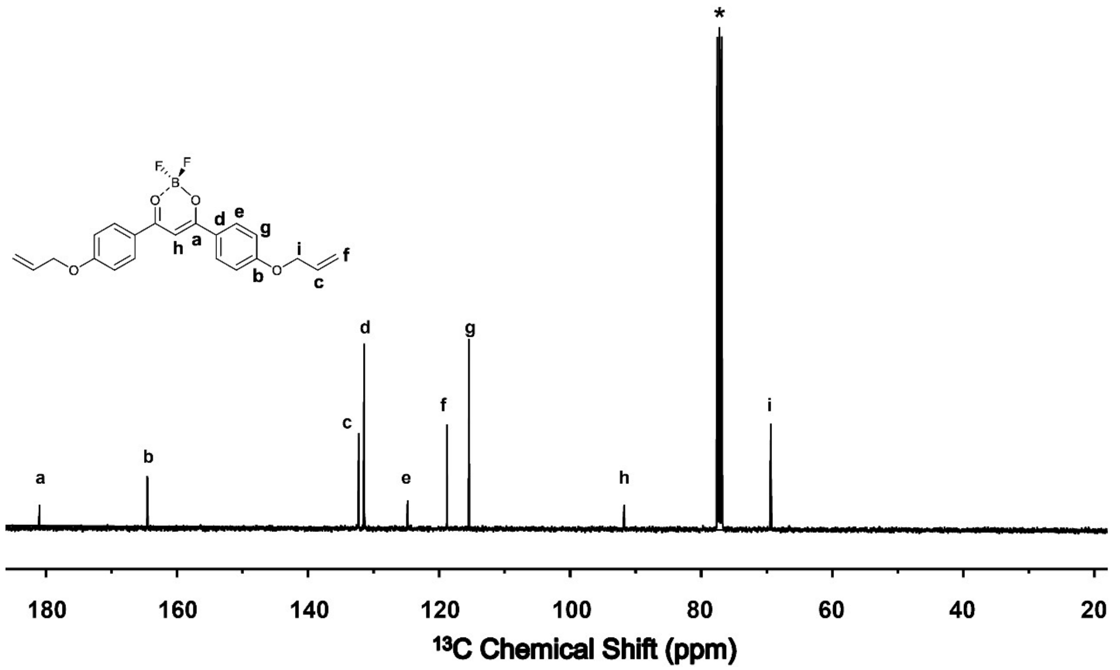

Figure S5. The ^{13}C NMR spectrum of BF_2bad in $CDCl_3$.

Table S1. Monomer proportions and yields of SPAP.

Sample	BF_2bad [mg]	AM [mg]	AIBN [mg]	DMSO [mL]	SPAP [mg]
SPAP(1/10)	250	463	6.4	4.5	300
SPAP(1/25)	100	463	6.4	4.5	350
SPAP(1/50)	50	463	6.4	4.5	450
SPAP(1/75)	33	463	6.4	4.5	410
SPAP(1/100)	25	463	6.4	4.5	470

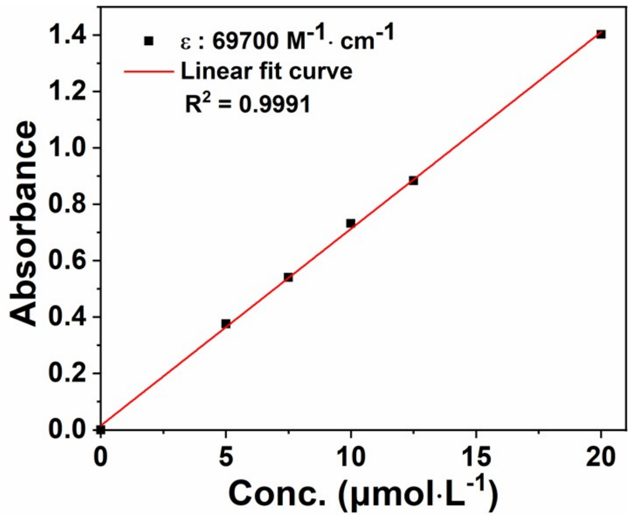

Figure S6. Molar extinction coefficient of BF_2bad in CH_2Cl_2.

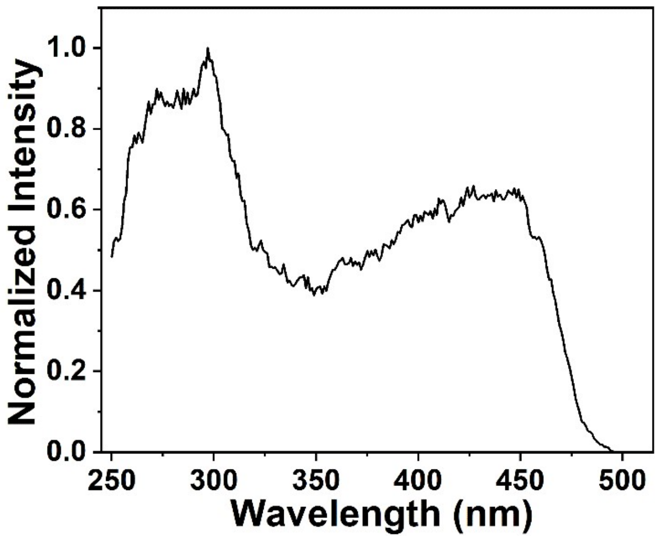

Figure S7. Excitation spectra of SPAP(1/50) (λ_{em} = 518 nm).

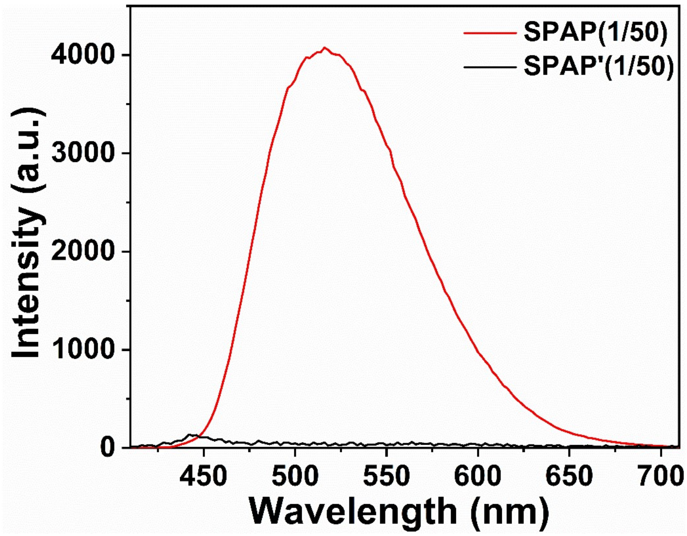

Figure S8. RTP emission spectra of SPAP(1/50) and SPAP'(1/50).

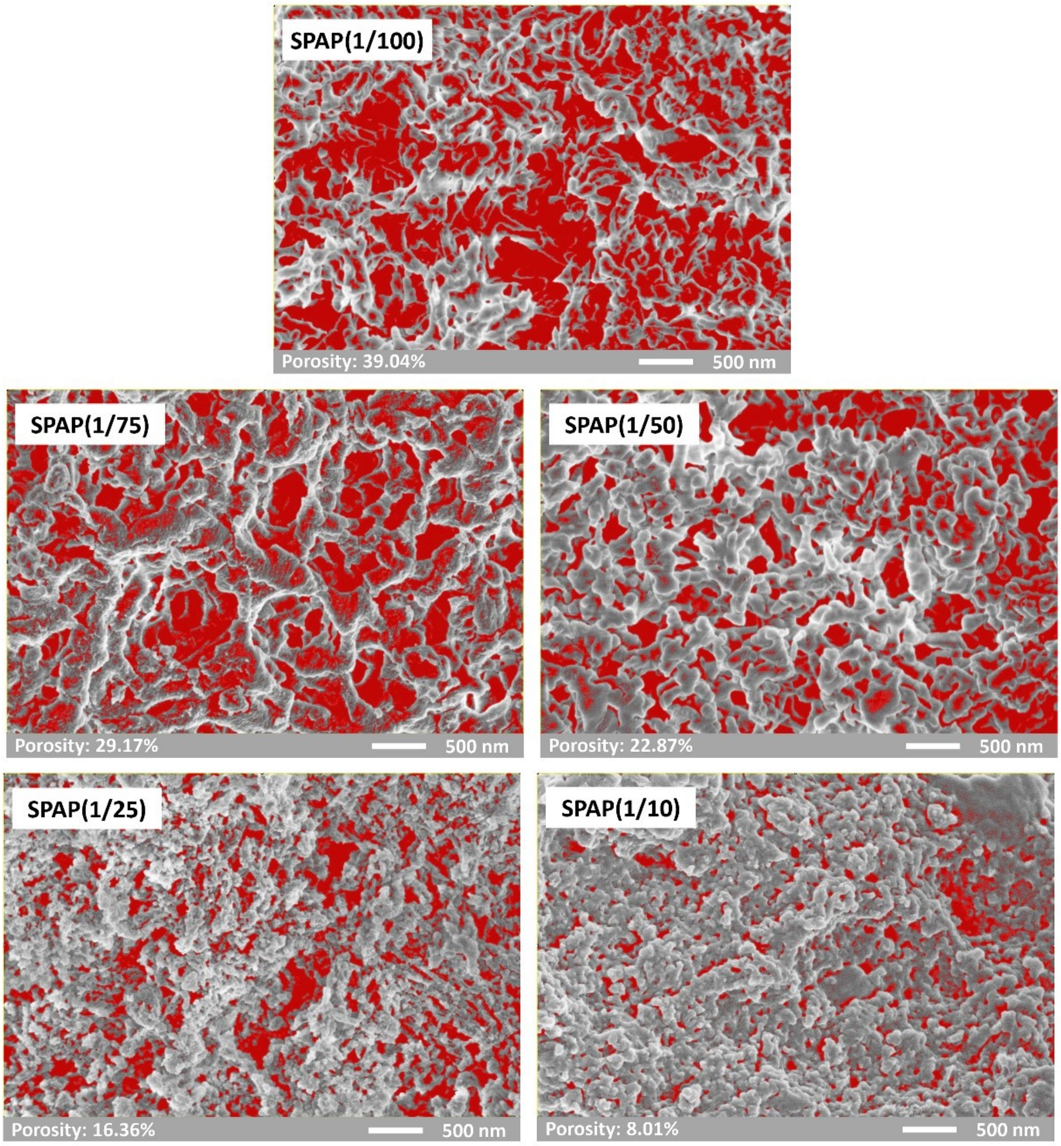

Figure S9. The estimated porosity of SPAP using ImageJ. The part as red color marked is the pore.

1. J. L. Howard, Y. Sagatov, L. Repusseau, C. Schotten and D. L. Browne, *Green Chem.*, 2017, **19**, 2798-2802.
